# Fluorescent Materials Based on Spiropyran for Advanced Anti-Counterfeiting and Information Encryption

**DOI:** 10.3390/molecules29112536

**Published:** 2024-05-28

**Authors:** Sha Ding, Xin Lv, Yong Xia, Yuejun Liu

**Affiliations:** Key Laboratory of Advanced Packaging Materials and Technology of Hunan Province, Hunan University of Technology, Zhuzhou 412007, China; 421298314@163.com (S.D.); 13100337389@163.com (X.L.)

**Keywords:** spiropyran-based fluorescent materials, multi-sensitivity, molecular switch, anti-counterfeiting

## Abstract

In daily life, counterfeit and substandard products, particularly currency, medicine, food, and confidential documents, are capable of bringing about very serious consequences. The development of anti-counterfeiting and authentication technologies with multilevel securities is a powerful means to overcome this challenge. Among various anti-counterfeiting technologies, fluorescent anti-counterfeiting technology is well-known and commonly used to fight counterfeiters due to its wide material source, low cost, simple usage, good concealment, and simple response mechanism. Spiropyran is favored by scientists in the fields of anti-counterfeiting and information encryption due to its reversible photochromic property. Here, we summarize the current available spiropyran-based fluorescent materials from design to anti-counterfeiting applications. This review will be help scientists to design and develop fluorescent anti-counterfeiting materials with high security, high performance, quick response, and high anti-counterfeiting level.

## 1. Introduction

Fake and inferior products, especially currency, medicine, food, and confidential documents, are counterproductive to the development of the global economy [1,2,3,4]. What is more serious is that they may also endanger national security and human physical and mental health. According to incomplete statistics, the global international trade in counterfeit goods was worth nearly USD 1 trillion in 2022 [5]. Despite the proliferation of overt and covert anti-counterfeiting techniques in recent decades, the persistent emergence of imitations remains a significant concern. Therefore, there is an urgent need to further improve anti-counterfeiting technology, to stop the flow of counterfeit products around the world.

Among the many anti-counterfeiting technologies, fluorescent anti-counterfeiting technology is well-known and most commonly used to against counterfeiters due to its wide material source, low cost, simple usage, good concealment, and simple response mechanisms [6,7,8,9,10,11,12]. Although extensive research has been conducted on the preparation of optimal fluorescent anti-counterfeiting materials, significant challenges still exist in achieving high-performance fluorescent materials with advanced anti-counterfeiting patterns [8,9,12,13,14,15,16,17,18]. Firstly, it is imperative to further develop a simplified, efficient, environmentally friendly, and controllable method for preparing fluorescent materials. Secondly, the ideal fluorescent anti-counterfeiting material should possess the following characteristics: minimal or colorless appearance under natural light, ability to emit intense and multicolored fluorescence under ultraviolet light, exceptional stability, and absence of biological toxicity, while meeting safety and environmental standards. Therefore, the selection of suitable materials is crucial for the development of fluorescent anti-counterfeiting materials that are both easy to identify and difficult to replicate.

**Figure 1:**
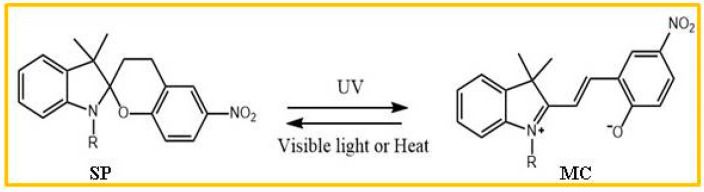
Structural diagram of spiropyran compounds.

Spiropyrans, a class of reversible photochromic compounds, have garnered significant attention and recognition in the field of materials science [19,20,21,22,23,24]. These compounds exhibit remarkable properties that make them highly promising for various applications. One notable characteristic of spiropyrans is their ability to undergo reversible photoisomerization between the closed-loop form (SP) and the open-loop form (MC) upon exposure to UV light and visible light (Figure 1). This means that they can switch between two distinct forms with different colors and chemical properties. Such versatility opens up possibilities for their use in optical devices, such as rewritable displays or data storage systems. Moreover, spiropyrans possess excellent photostability, allowing them to withstand prolonged exposure to light without degradation. This property makes them ideal candidates for long-lasting applications where durability is crucial. Additionally, spiropyrans demonstrate rapid response times during the photochromic transition process. Their ability to quickly switch between states enables high-speed modulation and control in optoelectronic devices like sensors or switches. Furthermore, these compounds exhibit good solubility in various organic solvents and polymers, facilitating their incorporation into different matrices or coatings. This compatibility enhances their potential utilization across diverse fields ranging from biomedical engineering to environmental monitoring. The unique combination of these advantageous characteristics positions spiropyrans as one of the most promising classes among known reversible photochromic compounds.

Ongoing research efforts continue to explore novel synthetic strategies and to optimize their properties further, aimed at unlocking even more exciting applications in areas such as smart materials or advanced photonics technologies. There are a number of studies reporting on the synthesis of spiropyran compounds [25,26,27,28,29], and applications of spiropyran-based materials in fluorescent probes [30,31,32,33,34,35], advanced photoswitchable materials [36,37,38,39,40], and bioimaging [41,42,43,44,45,46]. However, we found no review on the design of fluorescent materials based on spiropyran for anti-counterfeiting. In recent years, spiropyran and its derivatives have become more and more popular as the star molecules of fluorescence materials, and the preparation of fluorescence encryption and anti-counterfeiting materials using spiropyran and its derivatives has been widely reported. In this review, we will mainly focus on the recent progress in the design of spiropyran-based fluorescent materials and their anti-counterfeiting applications.

## 2. Application of Spiropyran in Fluorescent Anti-Counterfeiting and Information Encryption

As far as we know, the first literature review that mentioned the application of spiropyran and its derivatives in the field of anti-counterfeiting was completed by Kim and co-workers [47]. There have been many literature reports on the application of spiropyran-based fluorescence materials in the field of anti-counterfeiting. In the area of anti-counterfeiting applications, spiropyran fluorescent materials have extraordinary advantages when compared with other “advanced anti-counterfeiting and information encryption” materials. This superiority is demonstrated in their highly sensitive and specific response, which allows them to accurately identify and present under specific stimulations. At the same time, they have. excellent photochromic properties, enabling reversible changes in fluorescence and, thereby, effectively performing the anti-counterfeiting function. Furthermore, they are easily incorporated into various substrates and products.

Spiropyran fluorescent materials possesses the following advantages in aspects of data storage and information transfer [48,49,50,51,52,53]: (1) They exhibit excellent photochromic properties, capable of achieving the writing and erasing of information through diverse illumination conditions, thereby attaining repeatable data storage, and the storage density is relatively high. (2) Their fluorescent characteristics can offer unique signal identifiers, facilitating the accurate identification and transfer of information, and the stability of the signal is relatively good. (3) They feature a rapid response speed, being able to respond promptly to light stimuli, which is of great significance for efficient data storage and information transfer. (4) These materials can be combined or modified with other materials to adapt to different application requirements and environments, demonstrating strong adaptability and flexibility. (5) They are relatively stable and can maintain their performance for an extended period under certain circumstances, ensuring the reliability of data and information storage.

According to the composition of these fluorescent materials, they can be divided into organic small-molecule fluorescent materials, polymer fluorescent materials, metal–organic-framework fluorescent materials, nanoparticle fluorescent materials, etc. The following sections briefly discuss the encryption and decryption process of spiropyran-based fluorescent anti-counterfeiting materials in anti-counterfeiting applications.

### 2.1. Organic Small-Molecule Fluorescent Materials

Organic small-molecule fluorescent materials are favored by researchers due to their simple structure, readily available commercial raw materials, and ease of modification [54,55,56,57,58,59,60]. The spiropyran molecule is a typical small-molecule fluorescent material. Previous results have demonstrate that upon ultraviolet irradiation, the spiropyran molecule undergoes a transition from the unconjugated closed form (SP) to the conjugated merocyanine (MC) form, accompanied by an increase in molecular length and delocalized electrons. The MC isomer can be converted back to the SP form upon heating or exposure to visible light, requiring a large conformational freedom. However, the efficient conversion of SP compounds is one of the keys to the widespread application of this type of fluorescent material.

**Figure 2:**
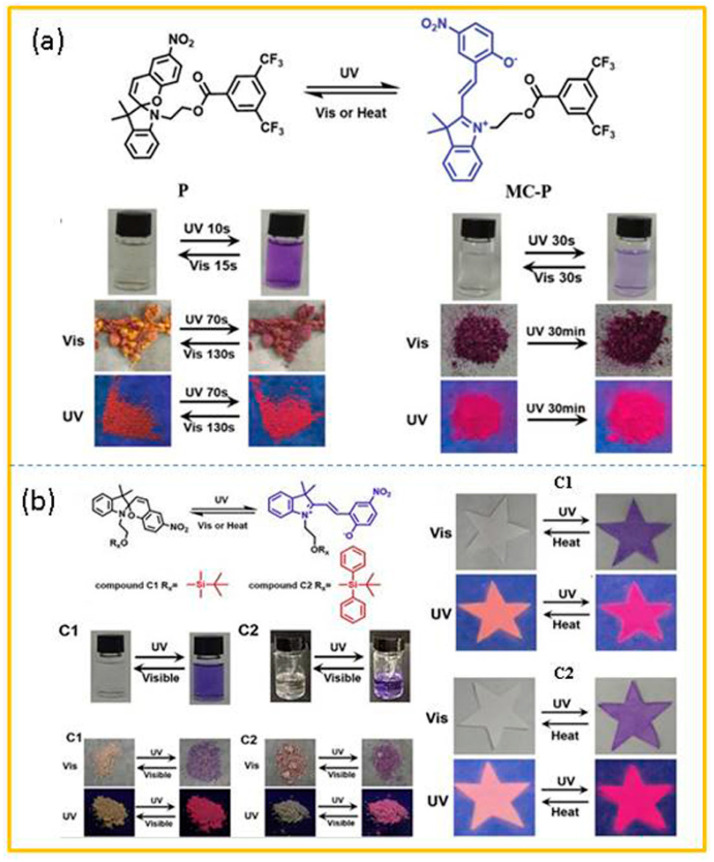
(**a**) The structure of compound P and its photochromic properties in solution and in the solid state. (**b**) The photochromic properties of C1 and C2 and their images as anti-counterfeiting ink on star-shaped filter paper under visible light and UV light. Reproduced from refs. [61,62].

He and co-workers [61] successfully enhanced the solid-state photochromism and mechanochromism of spiropyran by introducing a rigid hindrance group. The new photoswitching molecule (P) was synthesized by esterification of hydroxyl spiropyran (SP-OH) and 3,5-bis(trifluoromethyl)benzoic acid as raw materials. As shown in Figure 2a, under the alternating irradiation of ultraviolet and visible light, the new molecule showed highly reversible transformation in both solution and solid state, and had good fatigue resistance. Meanwhile, they replaced 3,5-bis(trifluoromethyl)benzoic acid with different silane groups, and synthesized photoswitching molecules C1 and C2 by the same method (Figure 2b) [62]. They made a star-shaped filter paper with a C1 and C2 solution (2 mg/mL). It was obvious that the star-shaped filter paper was colorless under visible light. Under 365 nm UV light, the star-shaped filter paper turned purple immediately, and could be distinguished by the naked eye. The purple star-shaped filter paper reverted to the original colorless state by heating at 70 °C for 10 min. What is more, the correlated fluorescence changes between pink and red radiation provided a higher contrast and clear signal. This was beneficial as a variety of color changes are conducive to the enhancement of the anti-counterfeiting level.

**Figure 3:**
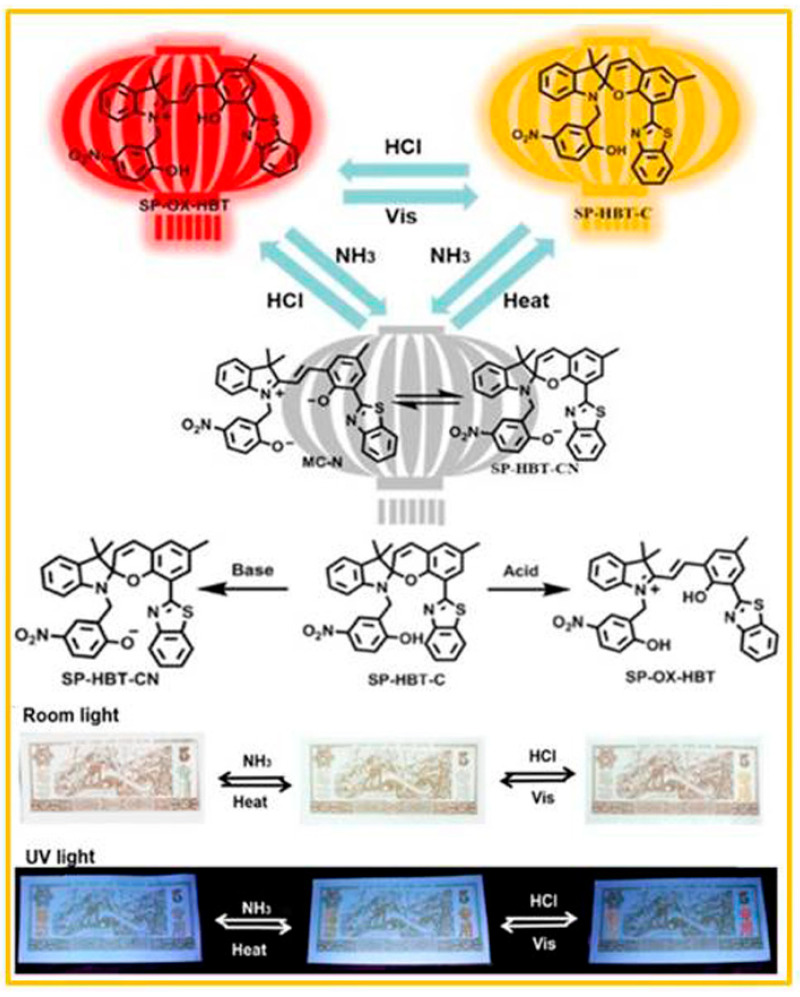
The transformation of the four fluorescent states of SP-OX-HBT and its anti-counterfeiting application under room light and UV light. Reproduced from ref. [63].

Liu and co-workers [63] designed a fluorescent molecular switch (SP-OX-HBT) with four color states in the solution and solid states, as shown in Figure 3. Under the action of visible light or a base, the conformation of SP-OX-HBT changed to the SP state (SP-HBT-C) or deprotonated SP state (SP-HBT-CN). The SP-HBT-C and SP-HBT-CN states had different visible and fluorescent colors. Significantly, solid SP-HBT-C powder had acid–base switching properties at room temperature. After base fuming, the SP-HBT-C state immediately transformed into the deprotonated MC form (MC-N), with the color of the powder changing from pale yellow to cyan. The MC-N, which had a broad absorption band at 630 nm, was stable in the solid state due to the restriction of conformational change. As shown in Figure 3, the two Chinese words, “lian gong” and “zhuan yong”, were printed on paper money for evaluation of the anti-counterfeiting effect. Under ambient conditions, the Chinese words were pale yellow. After HCl vapor fuming, the color of “zhuan yong” immediately turned orange. The color immediately changed back to its initial state with blue light irradiation. After NH_3_ vapor fuming, the color of “zhuan yong” turned to cyan and the color of the alkalized pattern changed back to pale yellow by heating to 80 °C.

**Figure 4:**
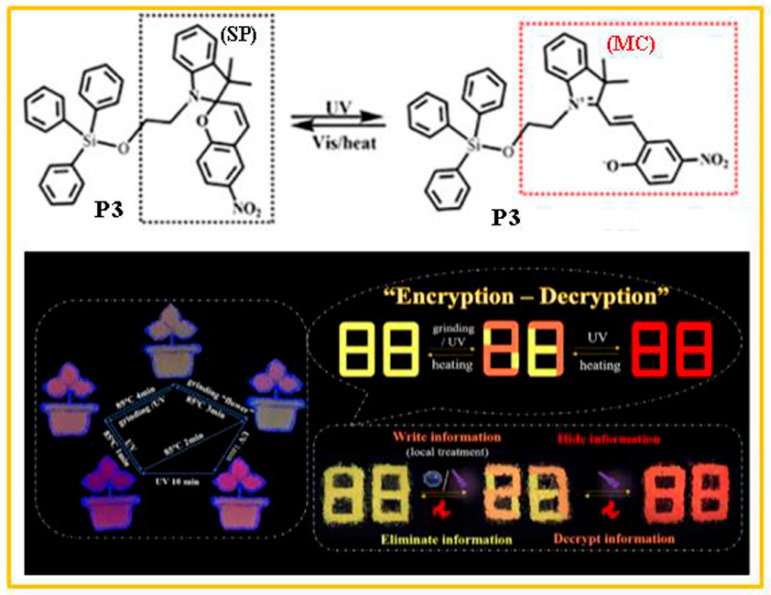
The structure of compound P3 and its multi-color switching process. Reproduced from ref. [64].

**Figure 5:**
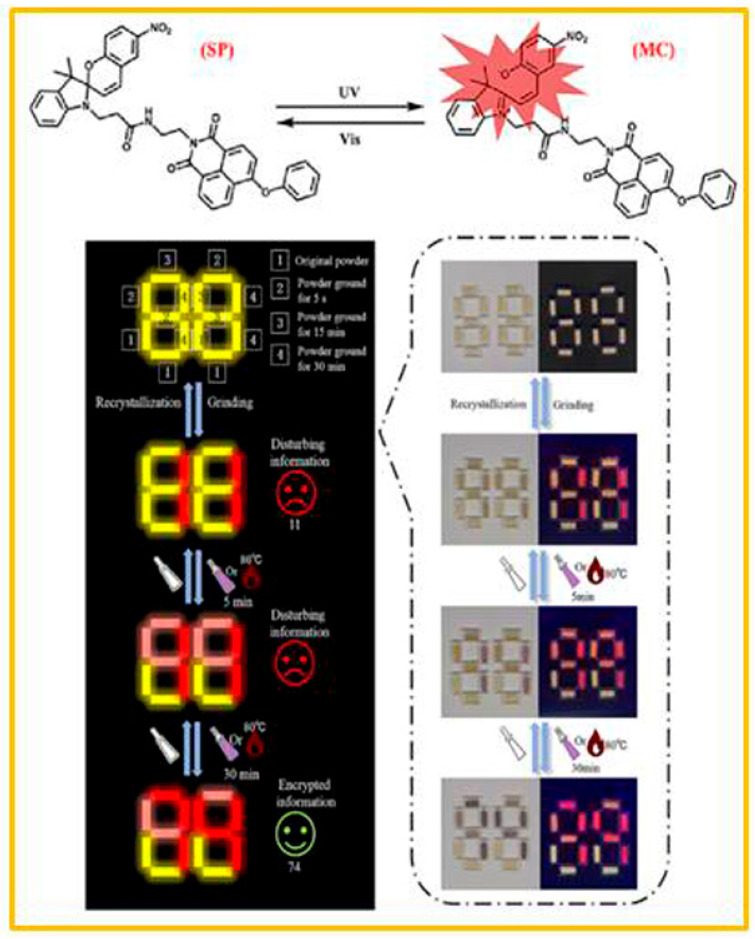
The structure of compound XG and the change in dual optical response to advanced information encryption and decryption. Reproduced from ref. [65].

Yang and co-workers [64] reported a new strategy to realize time-resolved encryption based on a silane-substituted spiropyran. The silane-substituted spiropyran (denoted as P3) was synthesized by substitution reaction of hydroxyl-containing spiropyran (SP-OH) and triphenylchlorosilane. Due to reversible photochromic and mechanochromic properties, the P3 had tunable dynamic photochromic properties with excellent reversible absorption/luminescence modulation capabilities in both powder and crystal states. As shown in Figure 4, the fluorescent pattern presented a “yellow flowerpot and orange flower” fluorescent pattern when the flower part was ground. The ring-opening isomerization of the flower part was greater than that of flowerpot because of grinding. Thus, the fluorescent patterns of “orange flowerpot and red flower” and “red flowerpot and red flower” appeared successively with the extension of ultraviolet-light time. Heating at 85 °C could restore the open ring mode of the P3 to different degrees, which were determined by the heating time. A yellow fluorescent “88” pattern was constructed by the P3 powder, the confidential information “27” was written on the paper by grinding or short-time UV irradiating. The “88” pattern appeared as red fluorescence when the whole body was exposed to UV light for 10 min, which hid the encrypted message temporarily. It is very convenient to see the encrypted information by heating it for a short time (85 °C for 2 min). Subsequently, a new spiropyran derivative (XG) was designed and synthesized via an amidation reaction between carboxyl-containing spiropyran (SP-COOH) and 4-phenoxy-N-ethylamino-1,8- naphthalimide (Ph-MH) [65]. Under different grinding conditions, this new compound exhibited distinct photochromic and thermochromic characteristics. Before UV irradiation, the fluorescence emission of the powder ground for 30 min was red, while the unground raw powder, the powder ground for 5 s, and the powder ground for 15 min were all yellow. After 5 min of UV irradiation, the powder ground for 30 min and the unground powder remained unchanged, but the powder ground for 5 s and the powder ground for 15 min both turned light pink. After 15 min of UV irradiation, compared with the 5 min UV irradiation, the powder ground for 30 min, the unground powder, and the powder ground for 5 s remained the same, while the fluorescence emission of the powder ground for 15 min turned red. In addition, the same effect as that caused by UV-radiation stimulation could be achieved by heating at 80 °C for different periods of time. Therefore, this new compound, XG, could be utilized to encrypt time-resolved information (Figure 5).

Spiropyran-based small-molecule fluorescent materials possess a multiplicity of characteristics. The advantages include having distinct photochromic properties in the aspect of optical performance, with conspicuously and sensitively alterable fluorescent colors; relatively good stability under specific conditions and the ability to maintain their performance; relatively low cost of preparation, with common raw materials, and a simple synthesis process; and good processability and the ability to be easily combined with other materials or manufactured into diverse forms. Nevertheless, there are also certain drawbacks. For instance, in terms of optical performance, there may exist a situation of relatively low fluorescence quantum yield; the stability may be susceptible to the influence of environmental factors such as temperature and humidity, thereby demonstrating a certain degree of decline; although the cost of preparation is not high, there is still scope for further reduction in cost in large-scale production; and in some complex application scenarios, the processability can encounter challenges and require further optimization and handling.

### 2.2. Fluorescent Polymer Materials

Traditional fluorescent polymer materials mainly comprise a polymer host and fluorescent dyes, where the fluorescent dyes are incorporated into the polymer host by physical mixing or chemical bonding [66,67,68]. Benefiting from the excellent photophysical properties of spiropyran fluorescent dyes, in recent years, an increasing number of researchers have incorporated them into the polymer matrix. This type of fluorescent material combines the excellent photophysical properties of spiropyran with the ease of processing of polymers, and is widely applied in biomedical, information, sensing, and other fields. This section mainly introduces their applications in anti-counterfeiting and information encryption in recent years.

**Figure 6:**
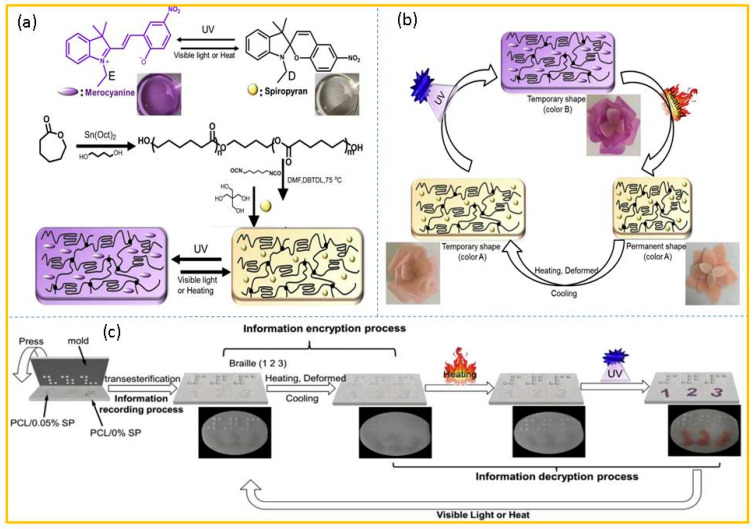
(**a**) Synthesis route of SP and PCL-N/SP networks. (**b**) The color change and shape change of the flower model under different stimuli. (**c**) The application of information-masking technology. Reproduced from ref. [69].

In 2019, Du and co-workers [69] fabricated a shape- and color-memory material (PCL-N/SP) by integrating spiropyran into the poly(ε-caprolactone) network. As shown in Figure 6a, the PCL-N/SP network was prepared via cross-linking reactions, employing a PCL precursor (NCO-PCL-NCO) as the primary constituent, tetrafunctional pentaerythritol as the cross-linking agent, and SP as the functional filler. Initially, the sample in a permanent shape (A, opened colorless flower) was deformed to a temporary shape (B, closed colorless flower) at a temperature higher than the melting point (80 °C) and fixed at a temperature lower than the crystallization temperature (0 °C). Subsequently, UV irradiation was employed to color the sample (C, closed amaranth flower). Therefore, a sample with different shapes and colors was obtained. Moreover, the bloom and fade process could be easily triggered by applying a proper external stimulus such as heating, and the rose flower was able to return to its original state, including shape and color. In other words, the whole process simultaneously realized shape memory and color memory (Figure 6b). Interestingly, this anti-counterfeit label, which utilized the dynamic bond exchange of urethane and ester bonds, not only helped to enhance the encryption level, but also, its decryption could also be recognized by the blind. First, the whole information recording process included the following two steps: embedding three digits “1”, “2”, and “3” (made of PCL-N/0.05% SP) into the blank substrate (made of pristine PCL-N) through bond exchange at 140 °C; and creating 3D embossed braille digital information on the substrate as a permanent shape by using a molding technique through a reconfiguration approach assisted by dynamic bond exchange. Afterwards, the information encryption process was completed by compression with a smooth plate at 80 °C and fixation at 0 °C. Ultimately, 3D braille was decoded through the shape memory recovery process by heating at 80 °C, and the corresponding 2D information was decoded by the color change of PCL-N/0.05% SP upon irradiation with 365 nm light (Figure 6c).

**Figure 7:**
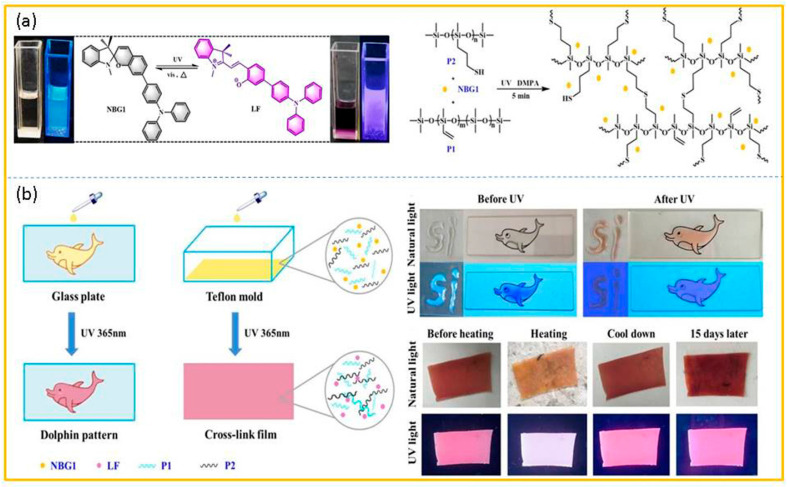
(**a**) The switch ring mechanism of NBG1 and the synthetic route of the silicone elastomer network. (**b**) The preparation of UV-stimulating reversible response film, Cns, and its fluorescence and color change under different stimuli. Reproduced from ref. [70].

Yang and co-workers [70] introduced a triphenylamine group to spiropyran to design a novel photochromic molecule (NBG1). Subsequently, novel photochromic polysiloxane-based films (Cns) were successfully fabricated using a simple thiol-ene ‘click’ reaction (Figure 7a). As shown in Figure 7b, after the UV radiation, the color and fluorescence of NBG1 showed alterations in the solution, and its fluorescence spectrum could also be regulated by controlling the solvent polarity and pH value. When constructed into a colorful thin film, the initial thin film was bright yellow. After 5 min of being irradiated by UV, the thin film in the shape of a dolphin became light pink and possessed corresponding red emissions. In addition, that thin film also presented temperature responsiveness. Further, the distinctive design and synthesis approach of Cns could be extended to numerous fields in the production of smart materials. Moreover, the QR code printed by NBG1 has performed dual protection in the anti-counterfeiting domain for expensive cosmetics and genuine medications, with high confidentiality.

**Figure 8:**
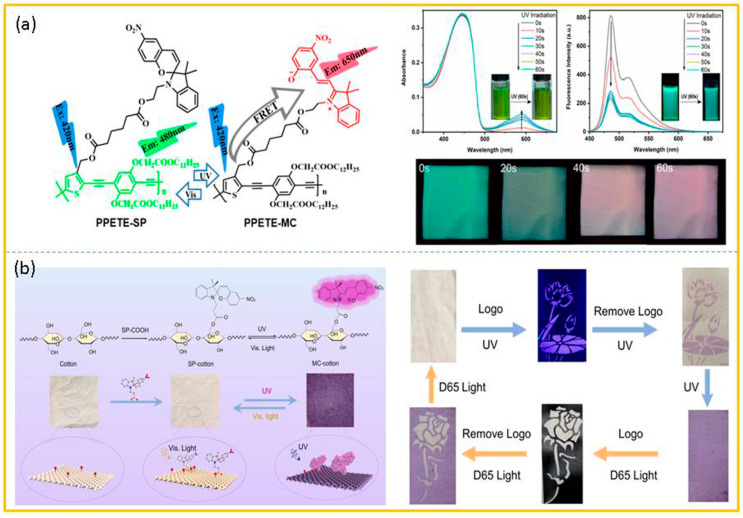
(**a**) The reversible transformation of PPETE and the change in its fluorescence color in solution and in a thin film. (**b**) The construction and photochromic mechanism of SP–cotton fabric and its anti-counterfeiting application. Reproduced from refs. [71,72].

Duan and co-workers [71] constructed a dynamic fluorescent anti-counterfeiting system by using poly[p-(phenylene ethynylene)-alt-(thienylene-ethynylene)] (PPTET) as the backbone and SP as the side chain. This system combined the photochromism triggered by the ring-opening of spiropyran (SP) with the fluorescence resonance energy transfer (FRET) among the conjugated polymer and MC (Figure 8a). Before ultraviolet irradiation, the absorption and emission of PPETE-SP presented both the curve of the typical PPETE framework, with the absorption peak around 446 nm, and the emission peak around 484 nm accompanied by a shoulder around 515 nm in the THF solution. After the ultraviolet-light irradiation, a new absorption peak appeared at 590 nm, and gradually increased with the irradiation time. However, surprisingly, as the irradiation time was increased, the emission intensity at 484 nm gradually decreased, and there was no appearance of new emission peaks at the long wavelength (unlike the emission derived from MC). Subsequently, the fibrous membrane of PPETE-SP was prepared by the method of electrospinning with PMMA as the matrix. With the increase in the UV light irradiation time, the fluorescent color of the membrane dynamically changed from green to light green, then to light pink, and finally to pink, and this process was reversible under visible light. Due to its excellent reproducibility and anti-fatigue property, this membrane could be directly used as an anti-counterfeiting label for multiple verifications. Gao and co-workers [72] constructed a durable and originally color-constant photochromic cotton fabric through the esterification reaction between SP and plant cotton. The stable ester covalent bond gave these SP-cotton fabrics proper washing fastness and light fatigue resistance. This kind of SP–cotton fabric was able to present the reversible transformation of colorless and purple under visible light and ultraviolet light. As depicted in Figure 8b, the area exposed by the photomask (lotus pattern) changed from colorless to purple under the ultraviolet-light irradiation, while the unexposed area retained its inherent colorlessness. In addition, this purple lotus could be converted into a white rose through switching the external light-source stimulus and different photomasks for long-distance control. This research could not only increase the added value of cotton, but it also provides us with a new simple and feasible idea for designing anti-counterfeiting materials.

**Figure 9:**
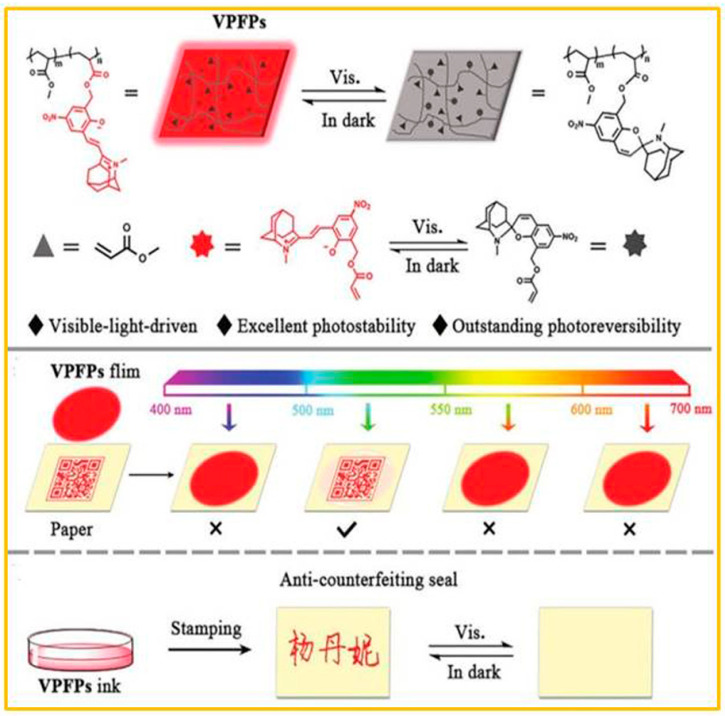
The design strategy of NSPMA and VPFPs, the corresponding photo-/thermo-induced isomerization mechanism of NSPMA, and the application of VPFPs in information encryption and anti-counterfeiting. Reproduced from ref. [73].

For the light stimulus, ultraviolet light was often used as the stimulus source, which would lead to inevitable photo bleaching and poor reversibility. In response to this problem, Li and co-workers [73] developed a visible-light-driven photoswitching fluorescent polymer, as shown in Figure 9. For this system, the author designed a new type of novel spiropyran monomer (NSPMA) with negative photochromic characteristics to achieve reversible fluorescence switching induced by visible light/heat, and chose poly (methyl acrylate) (PMA) as the matrix to promote its film-forming ability, light stability, and processability. The MC state of NSPMA presented intense red fluorescence; however, visible light (525 nm) caused isomerization to produce the unstable SP form with weak red fluorescence. These polymers significantly reduced the ACQ effect within a certain concentration range, thereby promoting the photostability of the material, and showed many appealing features, including high brightness, extraordinary photoreversibility, excellent photostability, and easy preparation.

### 2.3. Metal–Organic-Framework Fluorescent Materials

Metal–organic frameworks, abbreviated as MOFs, are a type of crystalline porous material that is formed through the interconnection of metal ions or clusters and organic ligands [74,75,76]. MOFs possesses high porosity and chemical stability, and combine the rigidity of inorganic materials with the flexible characteristics of organic materials. Incorporating it with spiropyran helps to enhance the comprehensive performance of the prepared fluorescent materials.

**Figure 10:**
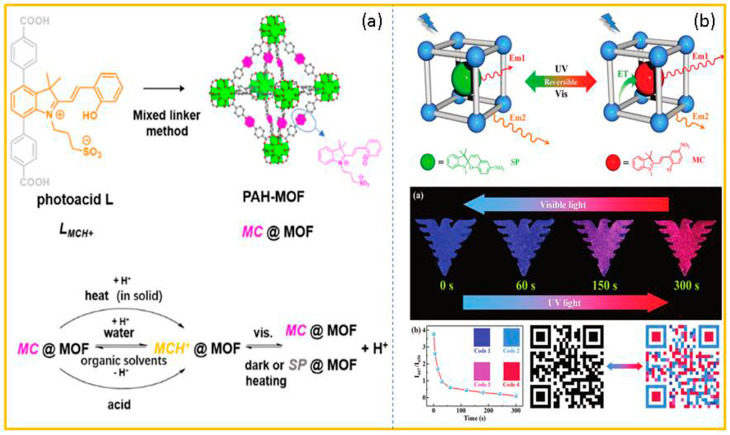
(**a**) The stimuli-responsive chromism of metastable-state photoacid. (**b**) The photo-stimuli-responsive dual-emitting luminescence of the ZJU-128⊃SP, and its application in information encryption and anti-counterfeiting. Reproduced from refs. [77,78].

Zhang and co-workers [77] prepared a multi-stimulus-responsive chromic material (PAH-MOF) by embedding a kind of metastable photoacid, that is, the protonated merocyanine form of spiropyran, into the zirconium(IV)-based UiO-topological metal–organic-framework material (MOF). PAH-MOF not only responded to light and heat, but also generated a stimulus response to different solvents and acids. Interestingly, PAH-MOF was not sensitive to light in the solid state, but the acidified PAH-MOF suspension exhibited excellent visible-light-induced photochromism in water and ethanol (Figure 10a). Zheng and co-workers prepared a novel photo-stimuli-responsive dual-emitting luminescent material ZJU-128⊃SP by encapsulating spiropyran molecules into a cadmium-based metal–organic framework (MOF) [78]. This fluorescent material exhibited blue emission derived from the ligand of ZJU-128 at 447 nm and red emission originating from spiropyran at 650 nm. When 365 nm ultraviolet light was used for continuous irradiation, the blue emission gradually became weaker, and the red emission gradually became stronger due to the photoisomerization of spiropyran and the intermolecular energy transfer. Interestingly, this dual-emission fluorescence switch could be regulated by visible light (Figure 10b).

**Figure 11:**
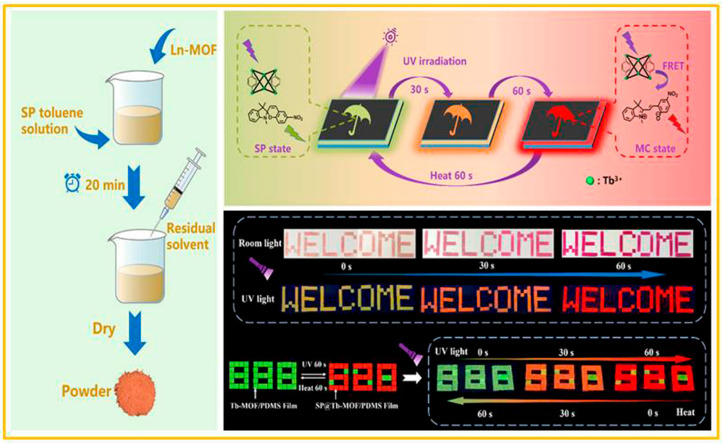
Schematic diagram for the preparation of fluorescent material SP@Ln-MOF, the mechanism of fluorescence change and images of the SP@Tb-MOF/PDMS film, and its information encryption and decryption. Reproduced from ref. [79].

Yang and co-workers [79] reported a simple and feasible method of building fluorescent materials through loading SP into a lanthanide-metal–organic framework. As depicted in Figure 11, the fluorescent material SP@Ln-MOF was obtained by soaking the Ln-MOF in the SP toluene solution in a dark environment for 20 min, then filtering out the solid, and then drying in a vacuum oven to remove the residual solvent. The results of 20 successive UV (365 nm, 60 s) irradiation and 10 min heat treatment cycles indicated that the SP@Ln-MOF composites had extremely good fatigue resistance. Based on this, they further constructed a fluorescent film (SP@Tb-MOF/PDMS) by doping the solid SP@Tb-MOF with a mass ratio of 0.5% into polydimethylsiloxane (PDMS). After stirring evenly in the glass bottle, it was dropped on the glass plate and heated at 90 °C for 4 h to solidify. When cooled to room temperature, the film was torn off the glass plate. Then, a beautiful pattern with high contrast could be accurately written on the film. As shown in Figure 11, the color of the umbrella changed from light yellow to pale pink after 30 s of ultraviolet-light irradiation, and then changed to purple with the extension of the irradiation time to 60 s, accompanied by the emission color having changed from yellow-green (SP state) to orange, and finally to red (MC state). Subsequently, they adopted a similar method to achieve the photochromism enhancement of spiropyran in the pretreated nanoporous lanthanide-metal–organic frameworks, and successfully applied it to information storage [80].

### 2.4. Nanoparticle Fluorescent Materials

Nanoparticle fluorescent materials, also known as nano fluorescent materials, are a type of advanced functional material [81,82,83]. Fluorescent nanoparticle materials have many excellent characteristics, such as very small size, high fluorescence efficiency, good stability, and a wide variety of applications [82,84,85]. The development of fluorescent nanoparticle materials has brought great potential and opportunities to scientific research and industrial applications. SP-based fluorescent nano-materials combining the outstanding properties of SP and nano-materials have already drawn the attention of researchers.

**Figure 12:**
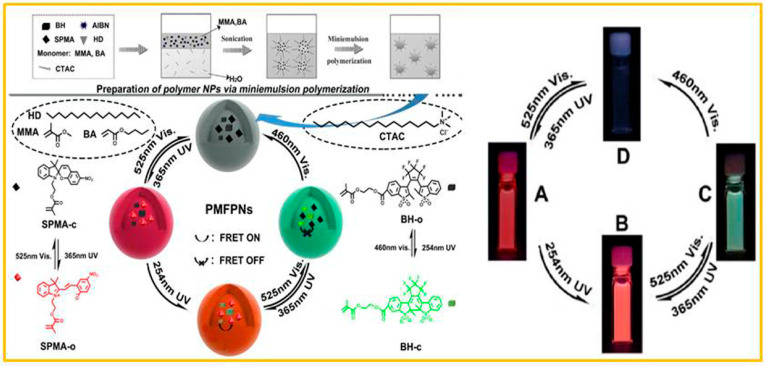
The preparation of polymer NPs and multi-wavelength regulation of the fluorescent state. Reproduced from ref. [86].

Owing to the switchable multiple emission states, and in comparison with the monochromatic or dual-color fluorescent systems, the photoswitchable multi-state fluorescent polymeric nanoparticles (PMFPNs) possess tremendous application potential in the domains of multi-layer information encryption, advanced optical anti-counterfeiting, and optoelectronic devices. Liu and co-workers [86] developed a category of novel multi-wavelength-regulated multi-state fluorescent polymeric nanoparticles by means of a facile one-pot miniemulsion polymerization. They made use of the photochromic fluorescent molecules diarylethene derivative (BH) and the spiropyran compound (SPMA), as well as the monomers methyl methacrylate (MMA), butyl acrylate (BA), hydrophobe (HD), an initiator (AIBN), and an emulsifier (CTAC), so as to obtain stable PMFPN dispersion through radical polymerization. As shown in Figure 12, the reversible transition of four fluorescent states was accomplished through adjusting the wavelength of the stimulating light. Before the ultraviolet-light irradiation, these two dyes were in the non-fluorescent state. After 365 nm (1 min) ultraviolet-light irradiation, the spiropyran unit changed from the closed-loop form (SPMA-c) to the open-loop form (SPMA-o), accompanied by the fluorescence emission from none to red. At this time, the BH unit had no structural change. After 245 nm (1 min) ultraviolet-light exposure, the structure of BH transformed from the open-loop form (BH-o) to the closed-loop form (BH-c). The fluorescence resonance energy transfer (FRET) between BH-c and SPMA-o caused the system to display orange fluorescence. When 525 nm (2 min) visible light was used for irradiation, due to SP changing into the closed-loop form, this FRET process was disrupted, and the system presented the green fluorescence of BH-c. After being exposed to 460 nm (3 min) visible light, the BH transformed into the open-loop form, and the fluorescence of the system went back to the initial non-fluorescent state. These nanoparticles possessed not merely fast photo-responsibility and splendid photo-reversibility, but also excellent thermal stability and long-term fluorescence stability. Sanjabi and co-workers also prepared switchable thermo- and photo-responsive polyacrylic nanocapsules by the miniemulsion polymerization of methyl methacrylate, in which the synthesized leuco-dye derivative (STC) and hydroxyl-functionalized spiropyran (SPOH) were simultaneously encapsulated [87]. Due to the synergetic effect of SPOH and STC, the thermochromic efficiency of the system was significantly improved, with an increased range of 1.8 to 2.3 times. In addition, multiple responses could be observed through UV-visible illumination, and the reflection peak shift caused the color to change from colorless to purple, and then to yellow.

**Figure 13:**
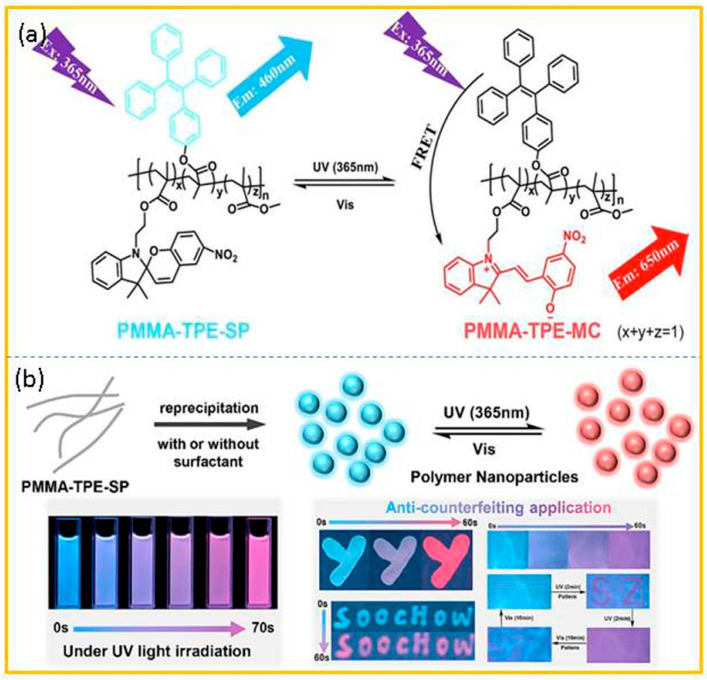
(**a**) The photoswitching FRET process between TPE and MC units in PMMA-TPE-SP. (**b**) The preparation of PNPs via reprecipitation and its anti-counterfeiting application. Reproduced from ref. [88].

Weng and co-workers [88] constructed a photoswitching dynamic color-changing fluorescent polymer nanoparticle system through fluorescence resonance energy transfer (FRET) between tetraphenylethene (TPE, a typical aggregation-induced emission structure) and spiropyran (SP, a photochromic structure). As shown in Figure 13a, the photochromic spiropyran and the aggregation-induced emission TPE were grafted onto the main chain of poly(methyl methacrylate) through covalent bonds in the photochromic polymer (PMMA-TPE-SP). Then, two types of fluorescent polymer nanoparticles, PMMA-TPE-SP (with no added surfactant (PMTS PNPs)) and PMMA-TPE-SP (with amphiphilic surfactant PSMA (PMTS/PSMA PNPs)), were prepared by the reprecipitation method (Figure 13b). The aqueous solution of polymer nanoparticles was concentrated by means of centrifugal ultrafiltration tubes. At the same time, a small amount of the nonionic surfactant Triton X-100 was added to avoid the aggregation of polymer nanoparticles during the centrifugation. Thereafter, an appropriate amount of glycerol and ethanol was added to the concentrated polymer nanoparticle solution so as to prepare the polymer nanoparticle ink. Afterward, a brush that had been pre-soaked with PMTS/PSMA polymer nanoparticle ink was used to write the corresponding pattern on the cellulose paper. After the ultraviolet-light irradiation, a change had occurred in the fluorescent color of the system. Along with the increase in the ultraviolet-light irradiation time, the fluorescent color of the system gradually changed from blue to light violet, and, finally, to red. The test results of the constructed double-layer anti-counterfeiting label showed that ultraviolet-light irradiation could make the pattern turn into pink, and the fluorescent color recovered after visible-light irradiation.

### 2.5. The Other Fluorescent Materials

**Figure 14:**
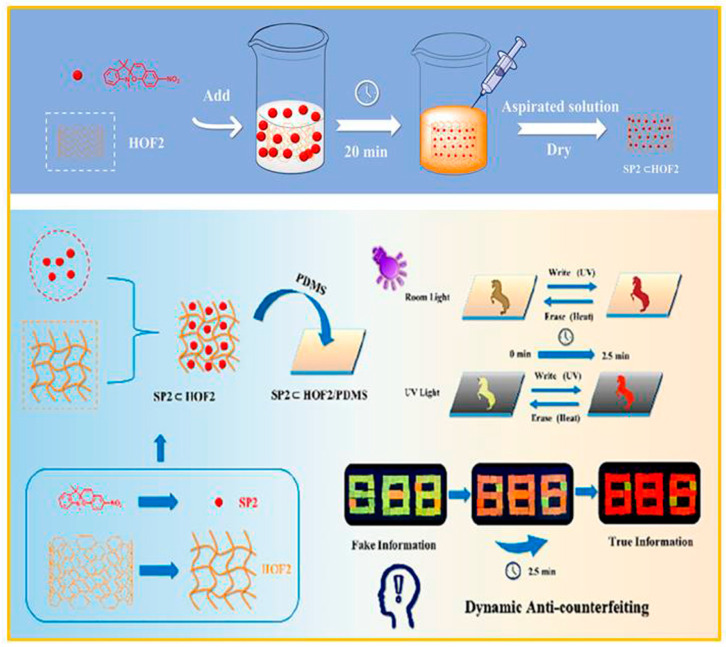
Schematic diagram for the preparation of fluorescent material SP2⊂HOF2, and its application in information encryption and anti-counterfeiting. Reproduced from ref. [90].

Organic hydrogen-bonded frameworks (HOFs) are a novel type of porous organic crystalline material, which is composed of organic molecules linked by non-covalent bonds (like hydrogen bonds, electrostatic interactions, and π-π stacking) [89]. Due to their advantages such as adjustable pore size, solvent processability, and easy recovery, HOFs have broad application prospects in catalysis, separation, photoelectricity, and biomedicine. Benefiting from the simple preparation process and adjustable fluorescent properties, the complex formed by SP and HOFs has received the attention of researchers. He and co-workers [90] presented a unique approach based on the combination of SP and HOFs, which allowed SP to have reversible switching in the solid state and presented a dynamic display of encrypted information. As shown in Figure 14, because of the fluorescence resonance energy transfer (FRET) process between the HOFs and the merocyanine (MC) isomer, the fluorescence emission underwent an evident transformation from yellow-green to orange to red with the extension of the irradiation time. The self-supporting film formed by doping with polydimethylsiloxane (PDMS) possessed high flexibility and mechanical strength, and could be bent and folded reversibly and repeatedly at angles of more than 90°. The introduction of HOFs greatly improved the fatigue resistance of the fluorescent material.

**Figure 15:**
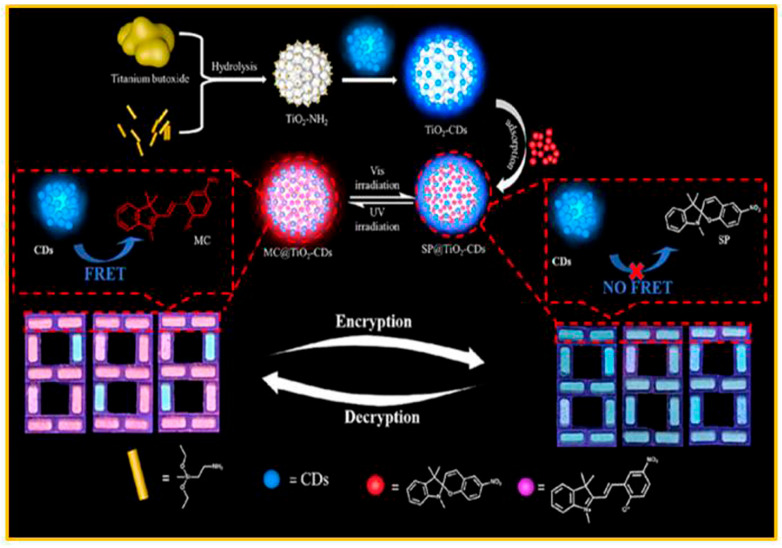
Schematic diagram for the preparation of SP@TiO_2_-CDs, and its application in information encryption. Reproduced from ref. [91].

Due to the features of low toxicity, biocompatibility, high light stability, and low cost, and the provision of multi-purpose luminescence characteristics, carbon dots (CDs) are widely used in anti-counterfeiting and information storage. The combination of CDs and SP provides a new idea for the high security of information encryption technology, especially time-dependent encryption. Through the combination of a photochromic spiropyran molecule and a titanium dioxide (TiO_2_)-grafted carbon dot (CD) system, a novel time-dependent information encryption technology was developed by Zhu and co-workers [91]. As shown in Figure 15, the color of the obtained composite materials could be reversibly switched between purple and colorless by irradiation with ultraviolet light and visible light. By controlling the isomerization of spiropyran units, the fluorescence resonance energy-transfer mechanism between the acceptor (SP) and the donor (CDs) could be regulated, thereby realizing the reversible ability of absorption/emission adjustment. With the gradual prolongation of irradiation time, a continuous and remarkable transformation was undergone by the fluorescence emission. It was transitioned from blue to pink and then, further, to red within a span of 20 min through the intricate process of fluorescence resonance energy transfer. During this entire process, various pieces of information, some of which might have been false while others were correct, were generated. Interestingly, the correct information could be identified at a specifically predesigned period of time, adding an extra layer of complexity and significance to the phenomenon.

## 3. Conclusions and Perspectives

This review presents a comprehensive analysis of the design and anti-counterfeiting applications of spiropyran-based fluorescent materials in recent years. The luminescence response behaviors towards various external stimuli including light, temperature, and mechanical force are introduced. We anticipate that future research will focus on development in the following areas, which will benefit the variety and application of spiropyran-based fluorescent materials: (1) The facile and controllable synthesis of spiropyran-based fluorescent materials. In fact, the synthesis and purification of spiropyran and its derivatives are difficult. During the preparation of spiropyran-based polymer fluorescent materials, the majority of which are random copolymers, there are difficulties in the exploration of the relationship between structure and performance. (2) Theoretical calculation, which may be a very effective and practical method of studying the responding mechanism of spiropyran-based fluorescent materials by using the density functional theory (DFT) and the time-dependent density functional theory (TDDFT). (3) Regulation of the excitation wavelength of spiropyran-based fluorescent materials. Currently, the excitation wavelength of spiropyran-based fluorescent materials is often limited to about 365 nm, which greatly limits their application scope. Therefore, it will be particularly important to develop near-infrared- or visible-light-activated spiropyran-based fluorescent materials with fast responsiveness, high photochromic efficiency, and photofatigue resistance (or optical durability). (4) Combining with intelligent response mechanisms, such as the coordinated response to various environmental factors such as temperature, humidity, and illumination, to enhance the diversity and intelligence of anti-counterfeiting performance. (5) Expanding the multi-modal anti-counterfeiting function of the materials, such as the integration of multiple characteristics such as fluorescence, magnetism, and color change.

In the future, the application of spiropyran fluorescent materials in multiple anti-counterfeiting functions will mainly focus on the following fields: intelligent packaging, to achieve unique anti-counterfeiting marks on high-end commodity packaging and provide real-time feedback on the authenticity status of commodities; and biomedical, which can be used for anti-counterfeiting of drugs or identification and authentication of medical equipment.
